# NMR data for the hydroxyl groups detected by NOESY spectra in sesquiterpene lactones

**DOI:** 10.1016/j.dib.2019.104246

**Published:** 2019-07-12

**Authors:** Jaromir Budzianowski, Joanna Nawrot, Gerard Nowak

**Affiliations:** a Department of Pharmaceutical Botany and Plant Biotechnology, Poznań University of Medical Sciences, Św. Marii Magdaleny Str. 14, 61-861 Poznań, Poland; b Department of Medicinal and Cosmetic Natural Products, Poznań University of Medical Sciences, Mazowiecka Str. 33, 60-623 Poznań, Poland

**Keywords:** Hydroxyl groups detection, NOESY spectra, NMR spectra, Sesquiterpene lactones

## Abstract

In this article we present the correlations observed in the NOESY spectra, which provide direct and unequivocal detection of hydroxyl groups occurring in the chemical structures of three sesquiterpene lactones - a germacranolide (8α-(*E*)*-*4′-hydroxysenecioyloxy-9α-hydroxyparthenolide) and two guaianolides (cynaropicrin and desacylcynaropicrin 8α-(*Z*)-(4′-hydroxy-2′-methyl)butenoate). The NOESY spectra and other NMR spectra, which served for the structural determination, are also presented. The data provided in this article are associated with the research article “Phytochemical profiles of the leaves of *Stizolophus balsamita* and *Psephellus sibiricus* and their chemotaxonomic implications” Nawrot et al., 2019.

**Table:** Specifications table

Subject area	*Phytochemistry*
More specific subject area	*Natural products research; structural characterization*
Type of data	*NMR spectra figures, structural formulae with NMR correlations arrows*
How data was acquired	*NMR spectra of sesquiterpene lactones acquired a Bruker Avance 600 spectrometer*
Data format	*Raw and analyzed*
Experimental factors	*First, the samples were isolated from CH*_*2*_*Cl*_*2*_*fractions of Psephellus sibiricus or Stizolophus balsamita leaf MeOH extracts.Then the samples were dissolved in aprotic solvents, like CDCl*_*3*_*and/or DMSO-d*_*6*_*before NMR studies.*
Experimental features	*Nuclear magnetic resonance (NMR) spectra were recorded on a Bruker Avance 600 instrument using* 600 MHz *and* 150 MHz *frequencies for hydrogen nuclei (*^*1*^*H) and carbon nuclei (*^*13*^*C), respectively, and tetramethylsilane (TMS) as internal standard. The aprotic solvents used contained residual water. The NOESY spectra were edited with different colours for the cross-peaks with different sign (negative, positive) to differentiate dipolar and chemical exchange interactions.*
Data source location	*Department of Medicinal and Cosmetic Natural Products, Poznań University of Medical Sciences, Mazowiecka Str. 33, 60–623 Poznań, Poland*
Data accessibility	*Data is provided in the article*
Related research article	Nawrot J, Budzianowski J, Nowak G. Phytochemical profiles of the leaves of *Stizolophus balsamita* and *Psephellus sibiricus* and their chemotaxonomic implications. *Phytochemistry* 159 (2019) 172–178 [1].

**Table:** 

**Value of the data** • The data are useful because they show that hydroxyl groups in sesquiterpene lactones can be detected directly by the use of a NOESY spectrum. • Researchers can benefit from this data during the structural elucidation of sesquiterpene lactones containing hydroxyl groups. • The data can be used whenever the NOESY spectra are utilized in the structural elucidation. • The additional value of the data is that in the case of NMR spectra recorded in CDCl3, the challenging detection of hydroxyl groups is avoided.

## 1 Data

**Fig. 1:**
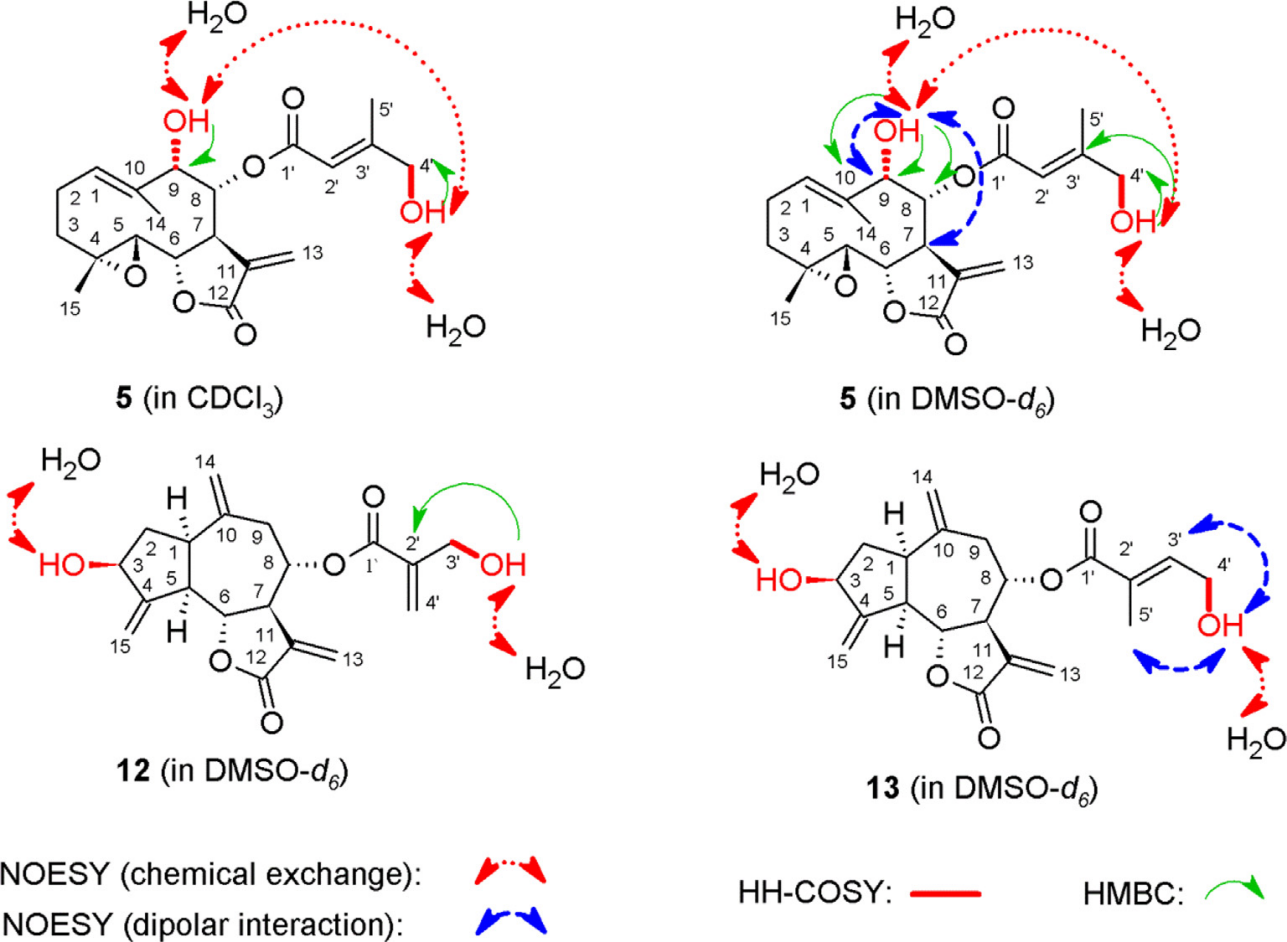
2D NMR - NOESY (dotted double arrows), HH-COSY (bold bonds) and HMBC (arrows) correlations observed for the hydroxyl groups signals of a germacranolide - 8α-(*E*)*-*4′-hydroxysenecioyloxy-9α-hydroxyparthenolide (compound **5**) and guaianolides – cynaropicrin (compound **12**) and desacylcynaropicrin 8α-(*Z*)-(4′-hydroxy-2′-methyl)butenoate (=cebellin F) (compound **13**). H_2_O refers to the solvent residual water. HSQC correlations are never observed.

**Fig. 2:**
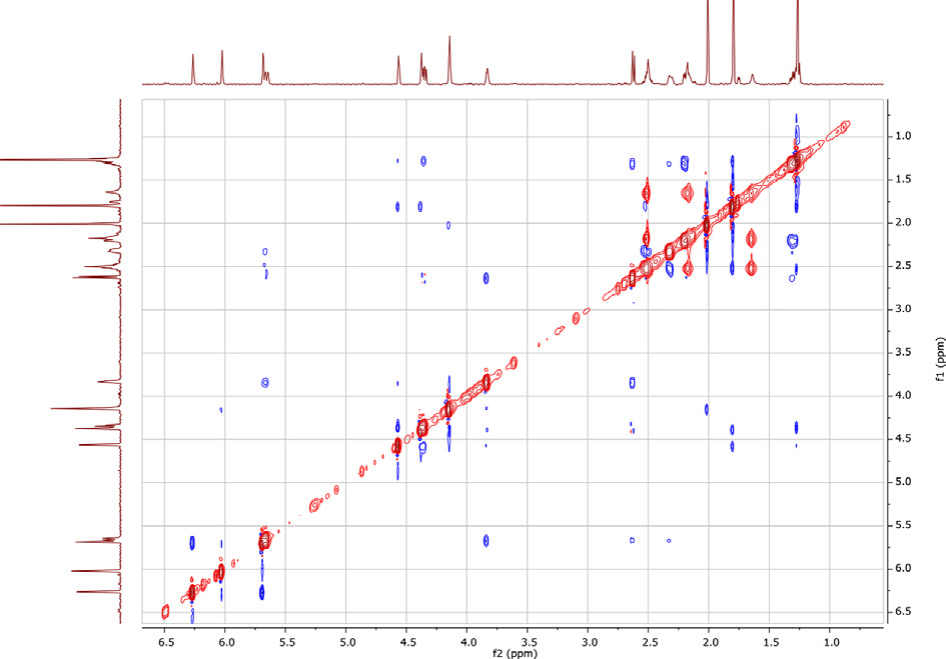
NOESY spectrum of compound **5** recorded in CDCl_3_. The red cross-peaks indicate signals of hydroxyl groups.

**Fig. 3:**
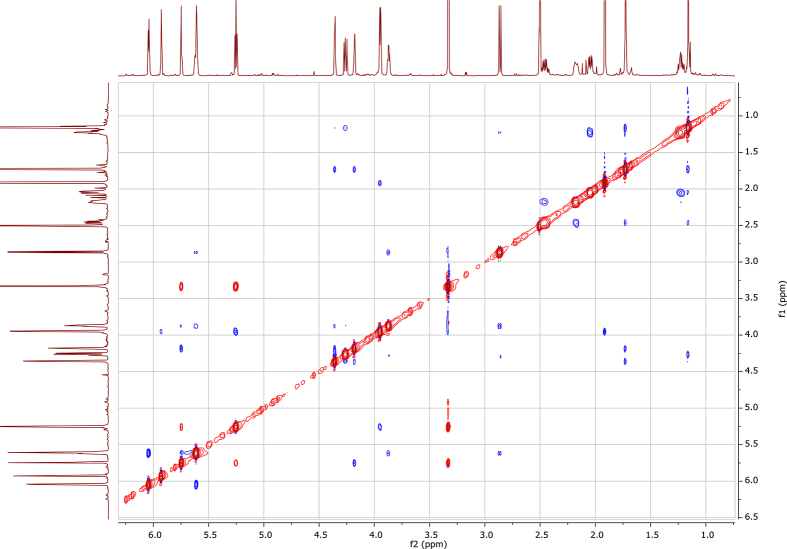
NOESY spectrum of compound **5** recorded in DMSO-*d*_*6*_. The red cross-peaks indicate signals of hydroxyl groups.

**Fig. 4:**
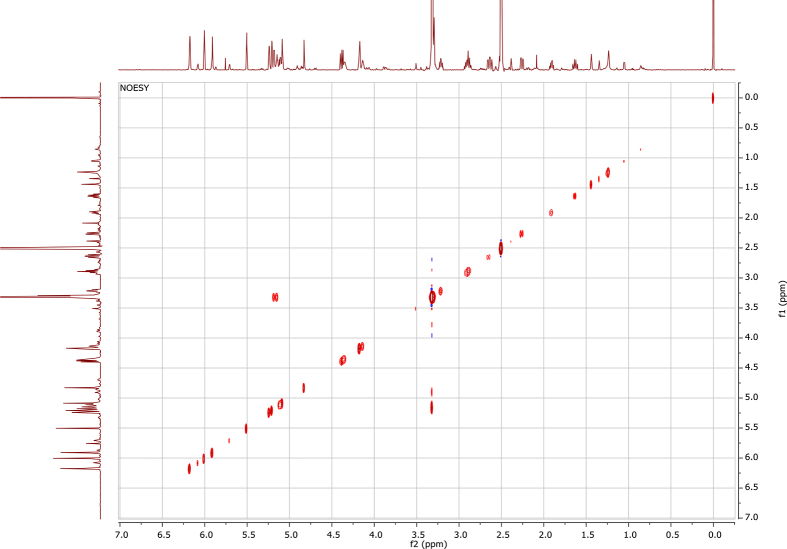
NOESY spectrum of compound **12** recorded in DMSO-*d*_*6*_. The intensity of the spectrum was tuned to show the chemical exchange cross-peaks (red) of the hydroxyl groups only.

**Fig. 5:**
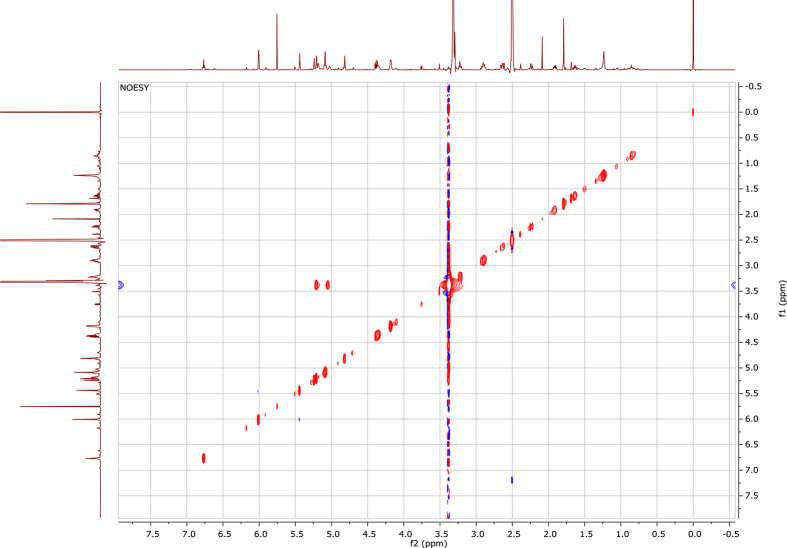
NOESY spectrum of compound **13** recorded in DMSO-*d*_*6*_. The intensity of the spectrum was tuned to show the chemical exchange cross-peaks (red) of the hydroxyl groups only.

**Fig. 6:**
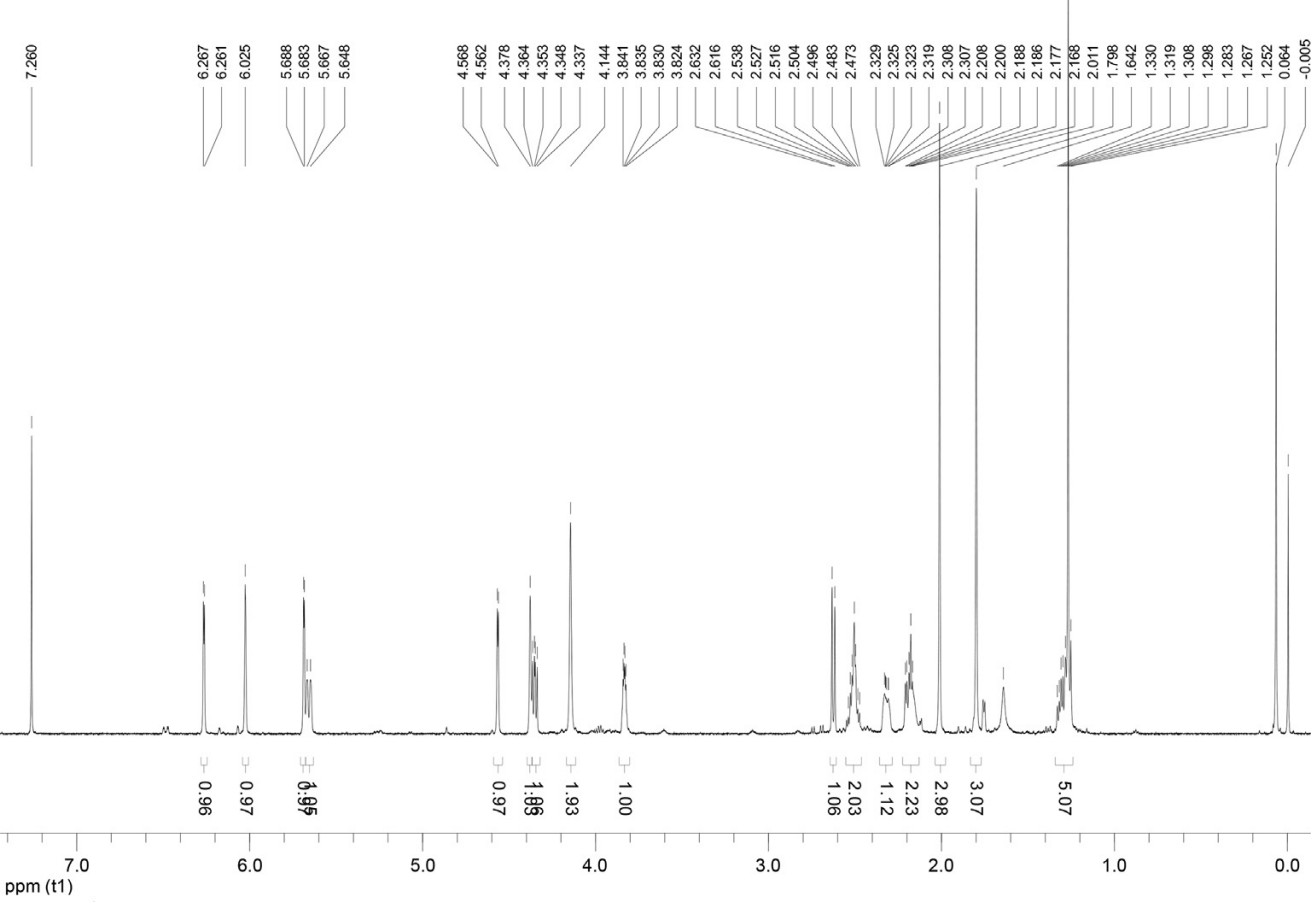
^1^H NMR (CDCl_3_) spectrum of compound **5**.

**Fig. 7:**
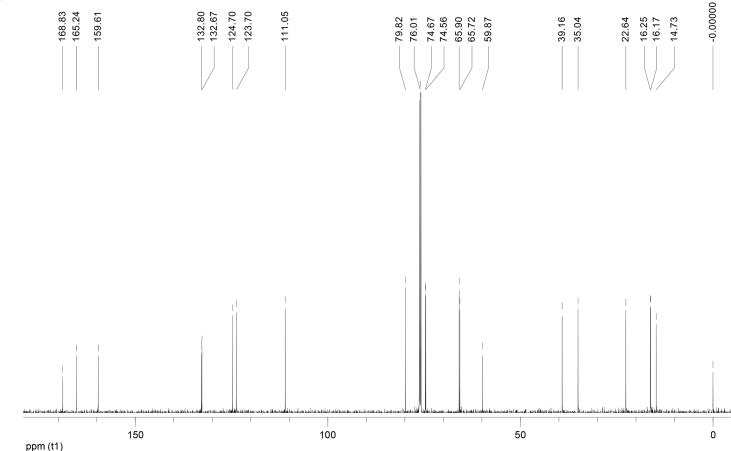
^13^C NMR (CDCl_3_) spectrum of compound **5**.

**Fig. 8:**
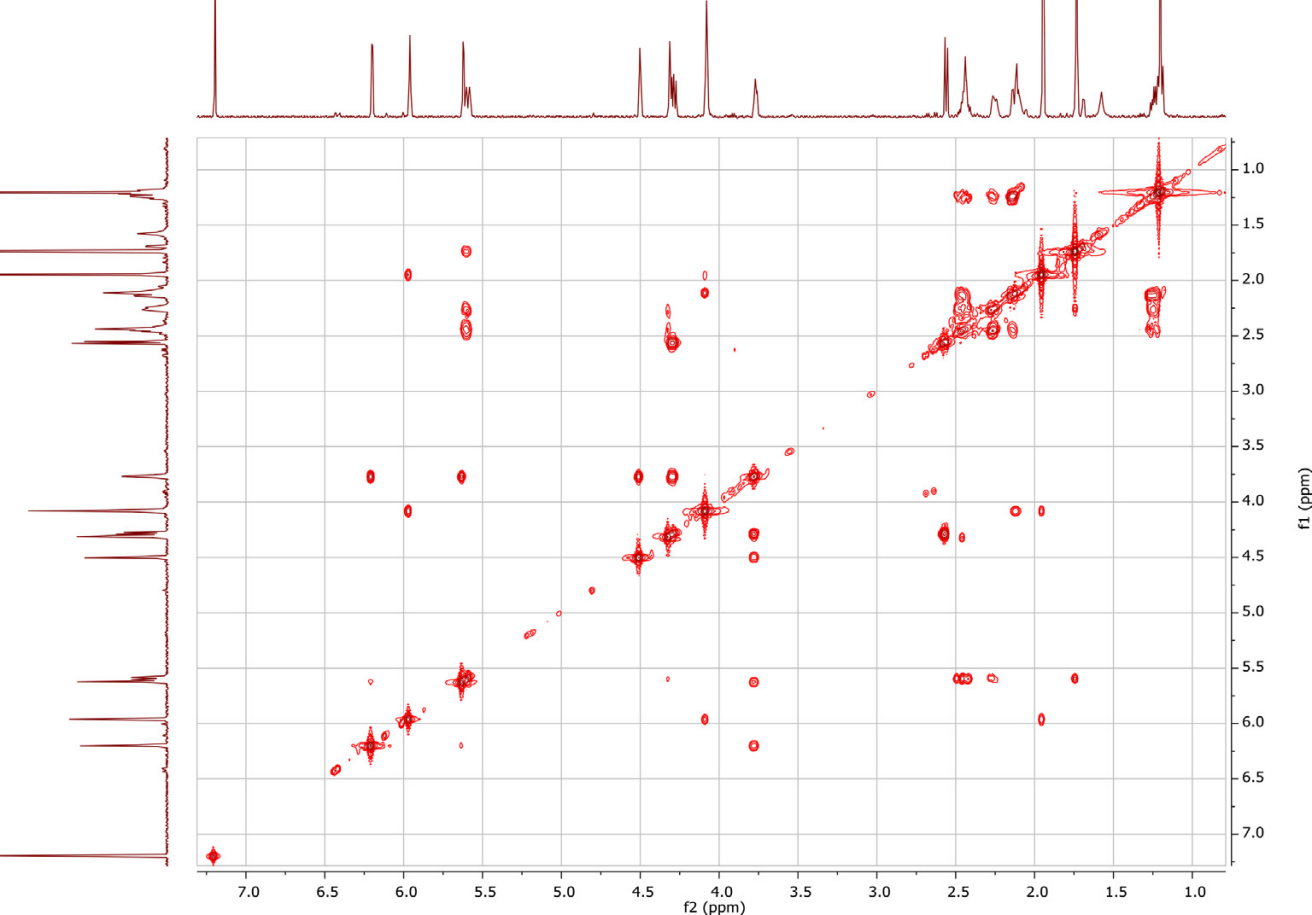
HH-COSY (CDCl_3_) spectrum of compound **5**.

**Fig. 9:**
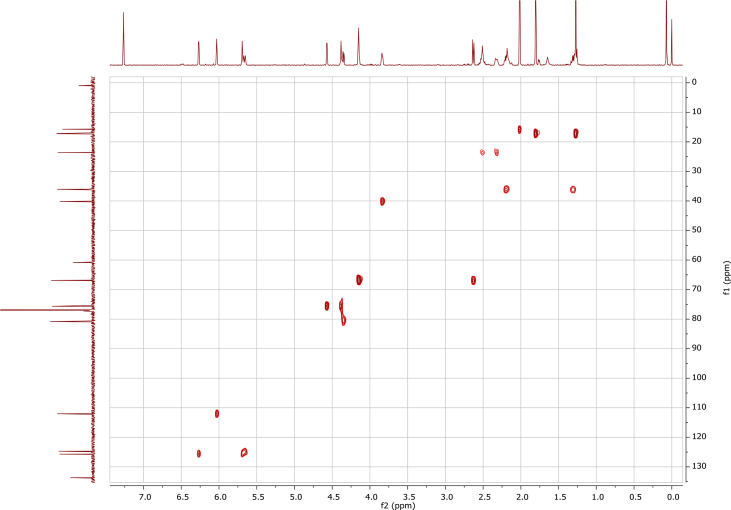
HSQC (CDCl_3_) spectrum of compound **5**.

**Fig. 10:**
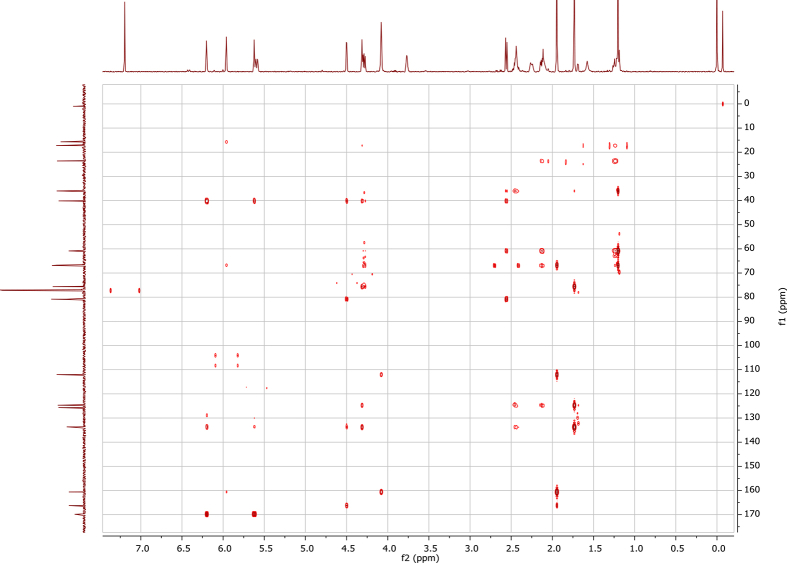
HMBC (CDCl_3_) spectrum of compound **5**.

**Fig. 11:**
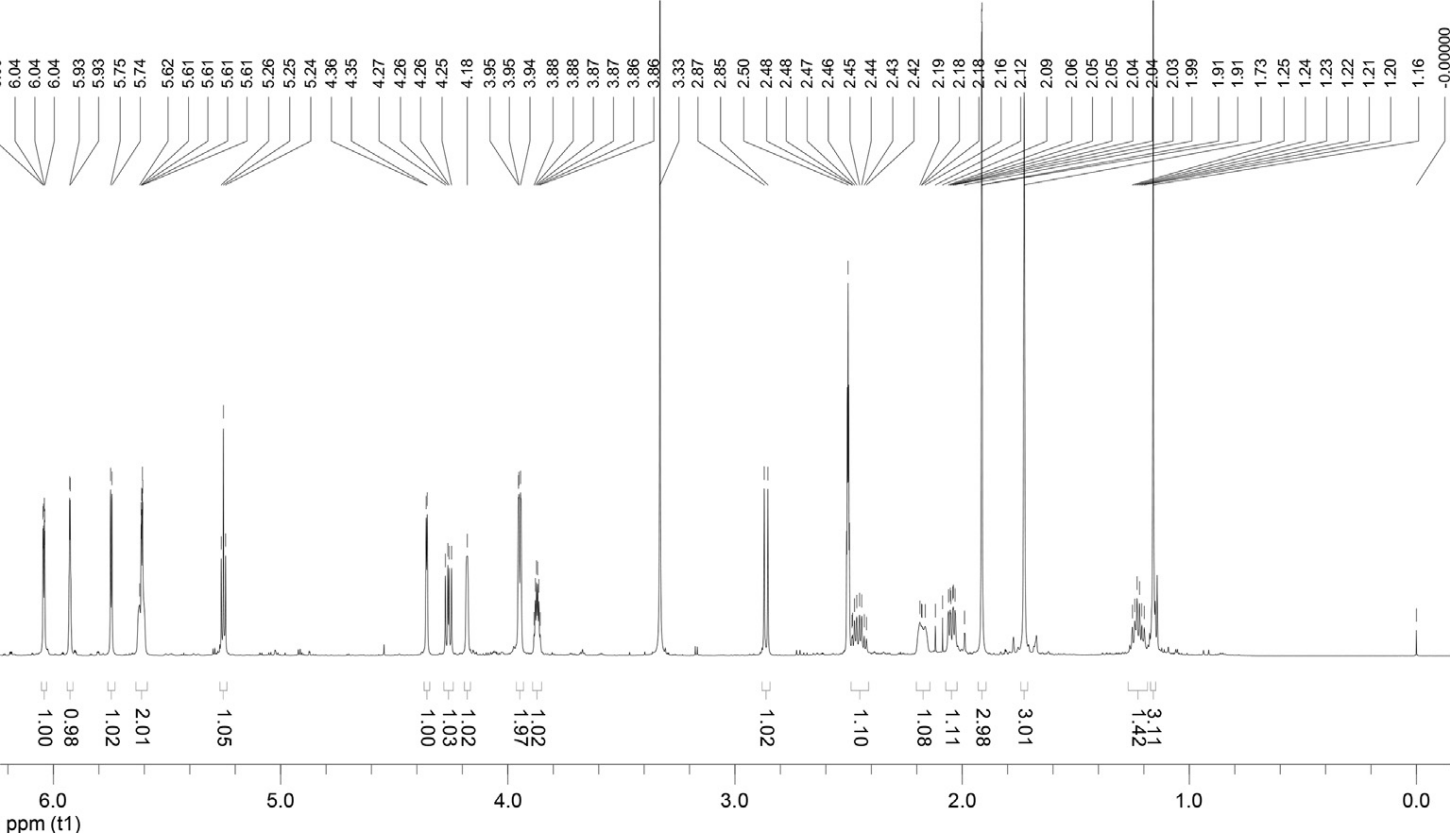
^1^H NMR (DMSO-*d*_*6*_) spectrum of compound **5**.

**Fig. 12:**
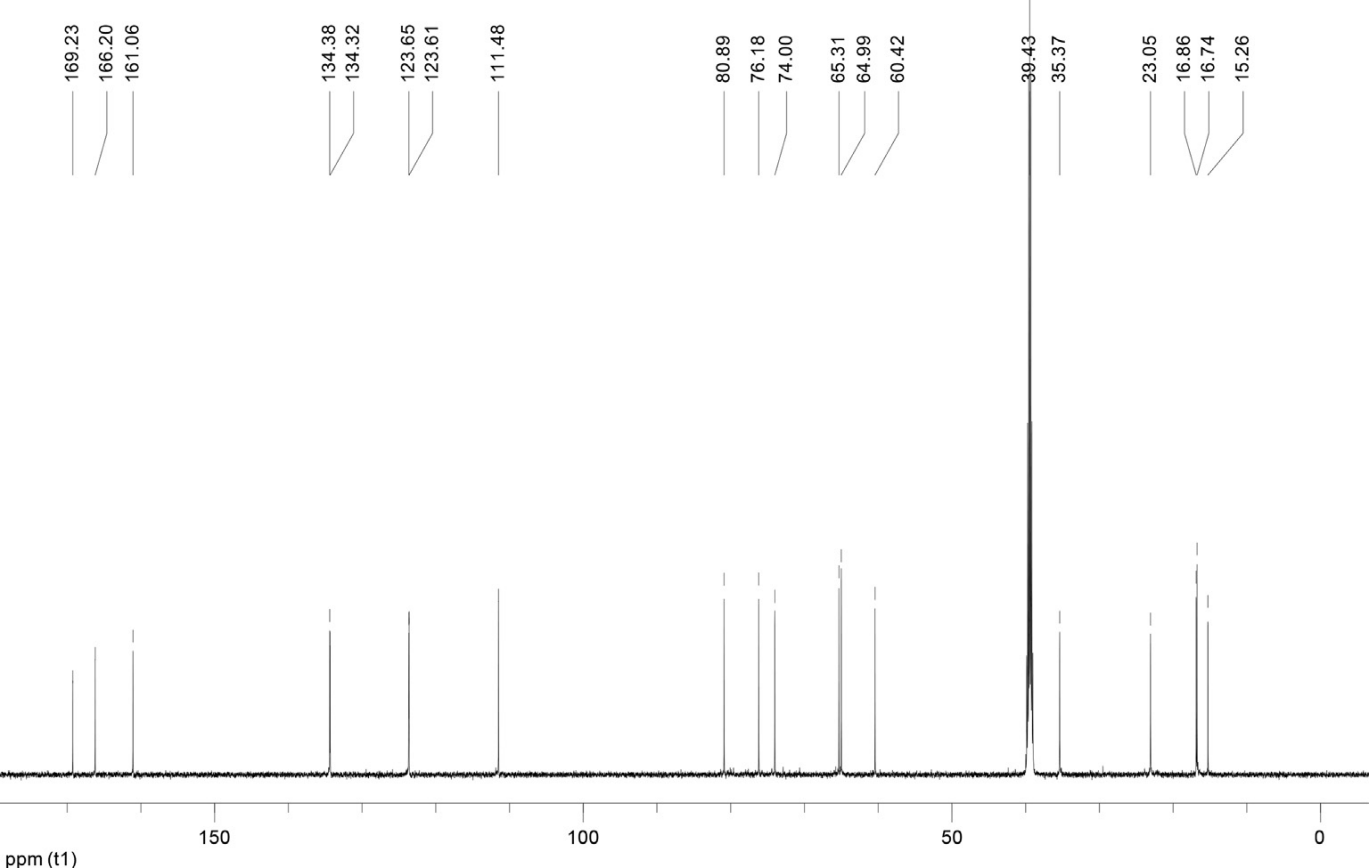
^13^C NMR (DMSO-*d*_*6*_) spectrum of compound **5**.

**Fig. 13:**
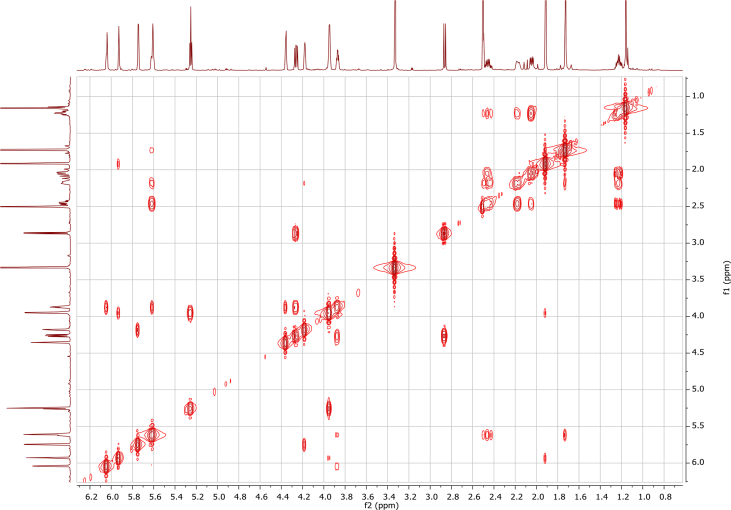
HH-COSY (DMSO-*d*_*6*_) spectrum of compound **5**.

**Fig. 14:**
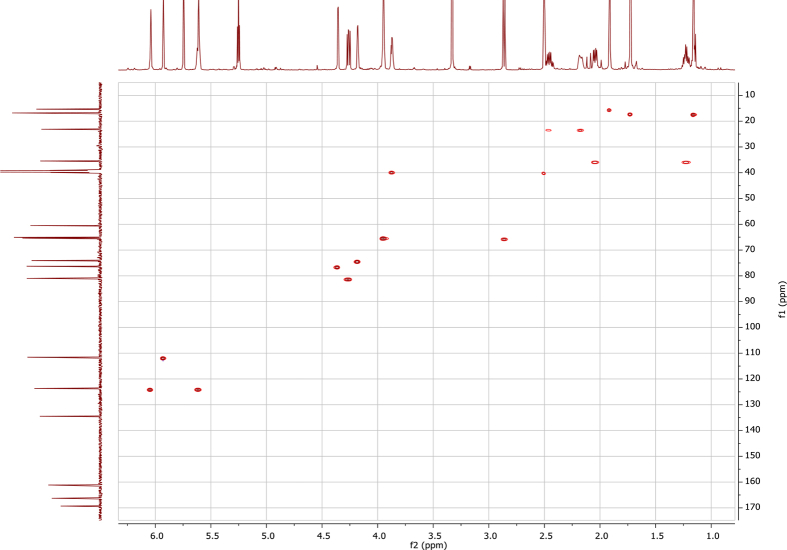
HSQC NMR (DMSO-*d*_*6*_) spectrum of compound **5**.

**Fig. 15:**
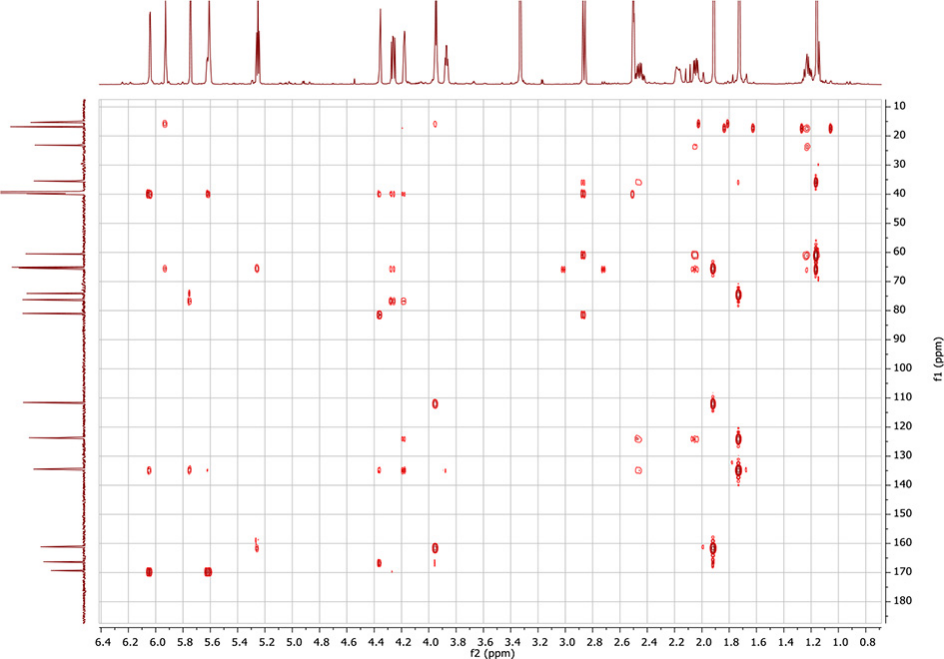
HMBC (DMSO-*d*_*6*_) spectrum of compound **5**.

**Fig. 16:**
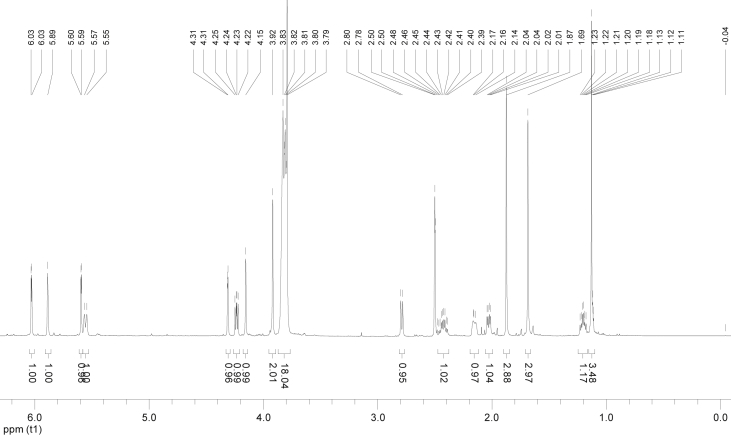
^1^H NMR (DMSO-*d*_*6*_+ D_2_O) spectrum of compound **5**.

**Fig. 17:**
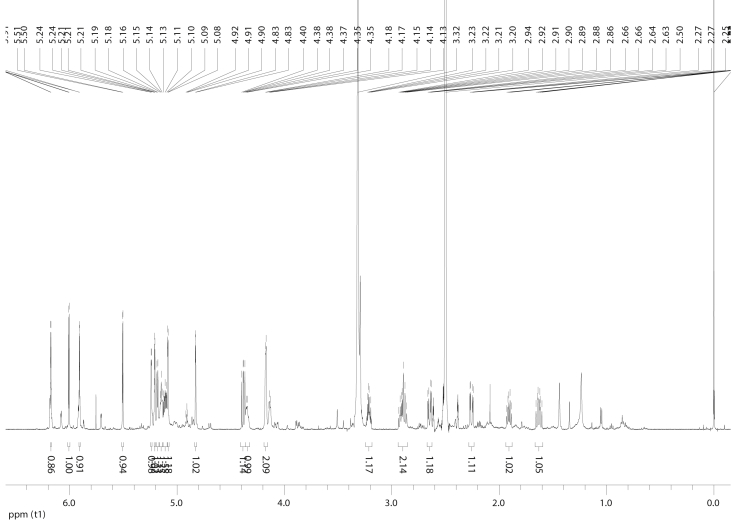
^1^H NMR (DMSO-*d*_*6*_) spectrum of compound **12**.

**Fig. 18:**
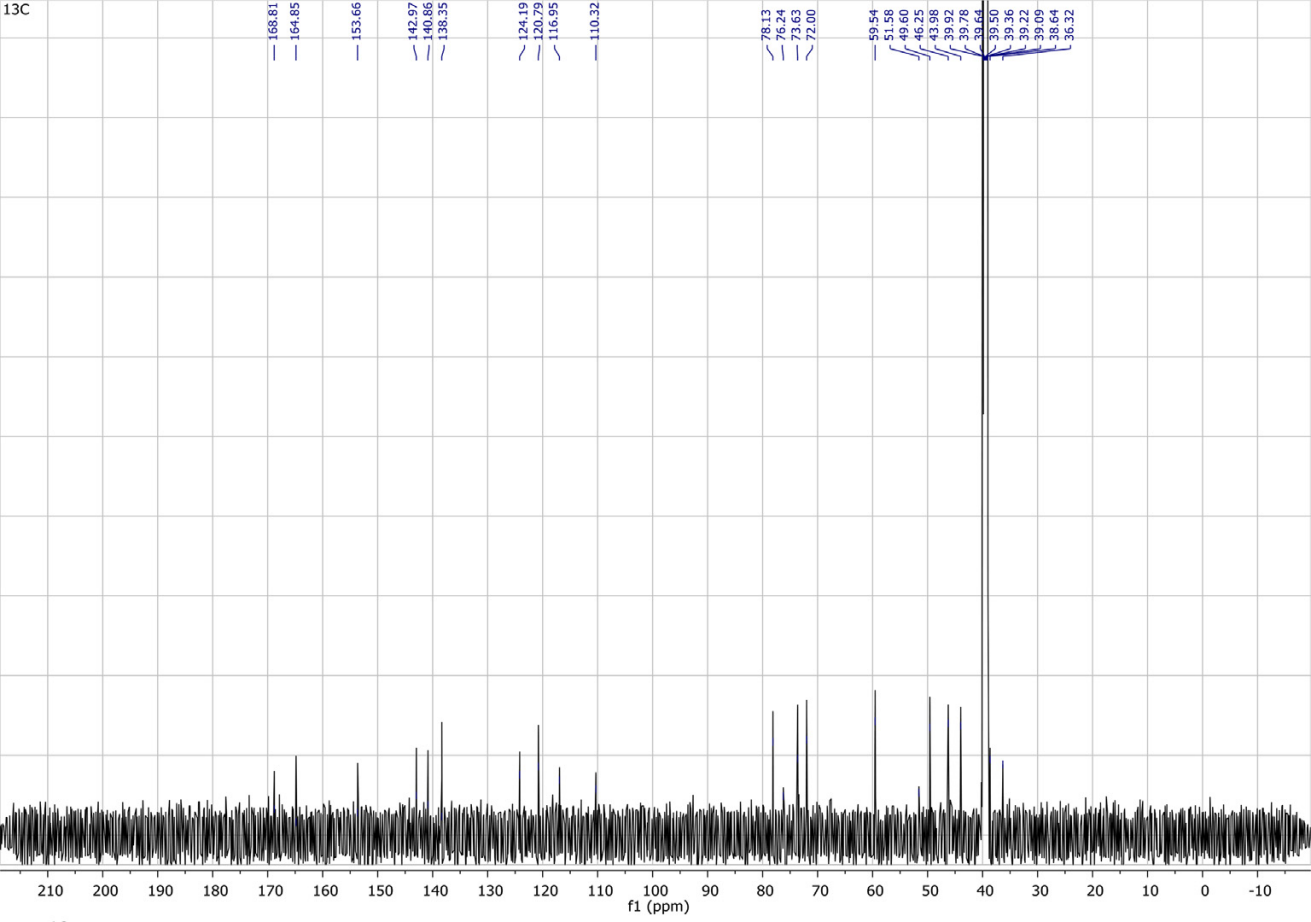
^13^C NMR (DMSO-*d*_*6*_) spectrum of compound **12**.

**Fig. 19:**
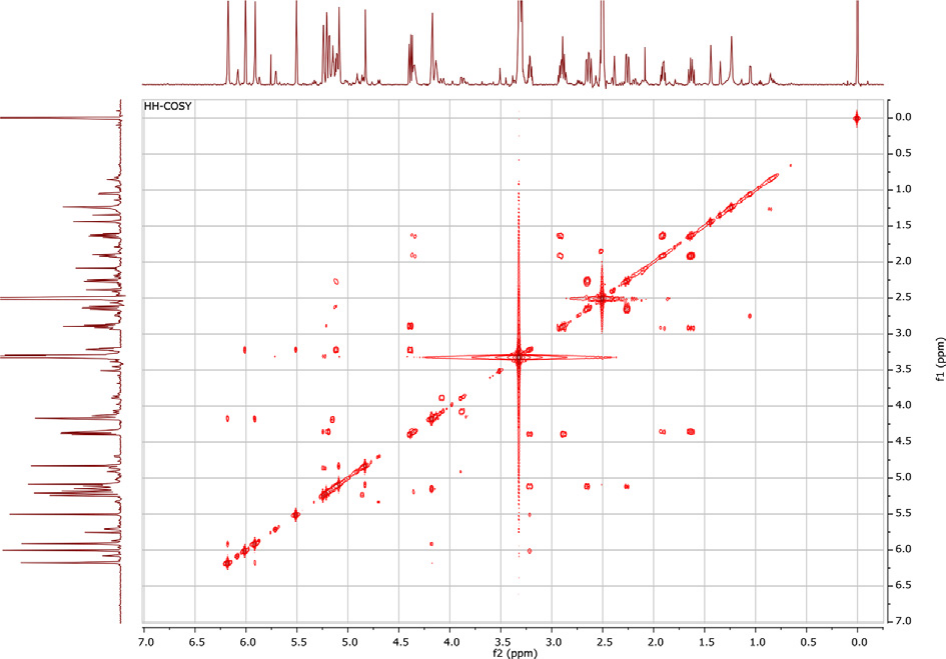
HH-COSY (DMSO-*d*_*6*_) spectrum of compound **12**.

**Fig. 20:**
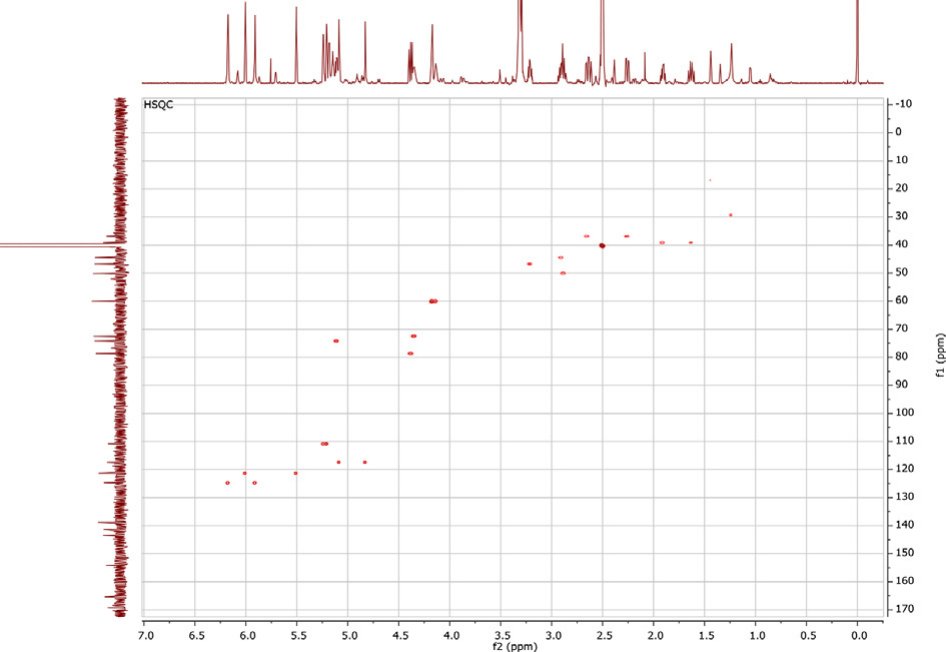
HSQC (DMSO-*d*_*6*_) spectrum of compound **12**.

**Fig. 21:**
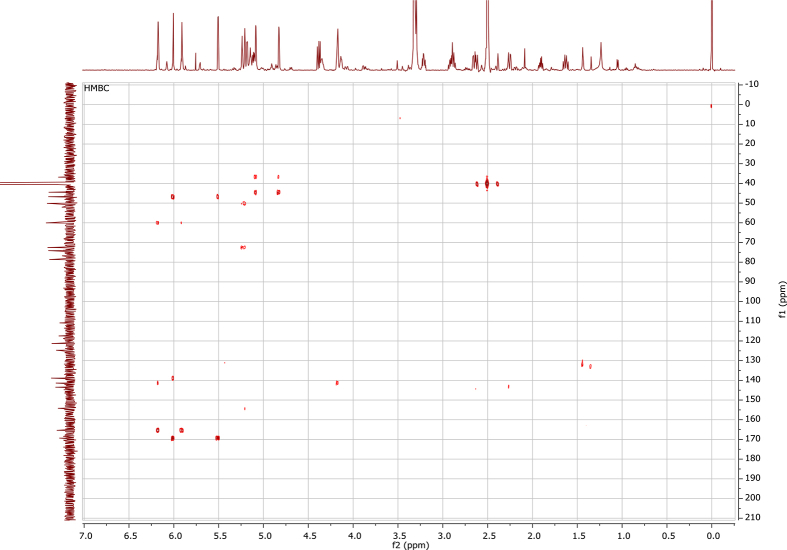
HMBC (DMSO-*d*_*6*_) spectrum of compound **12**.

**Fig. 22:**
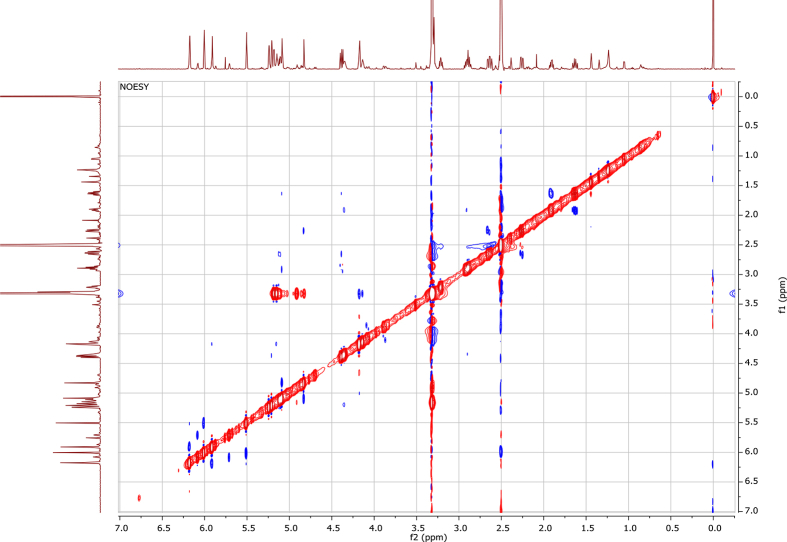
NOESY (DMSO-*d*_*6*_) spectrum of compound **12**. The intensity of the spectrum was tuned to show the cross-peaks due to the dipolar couplings (blue).

**Fig. 23:**
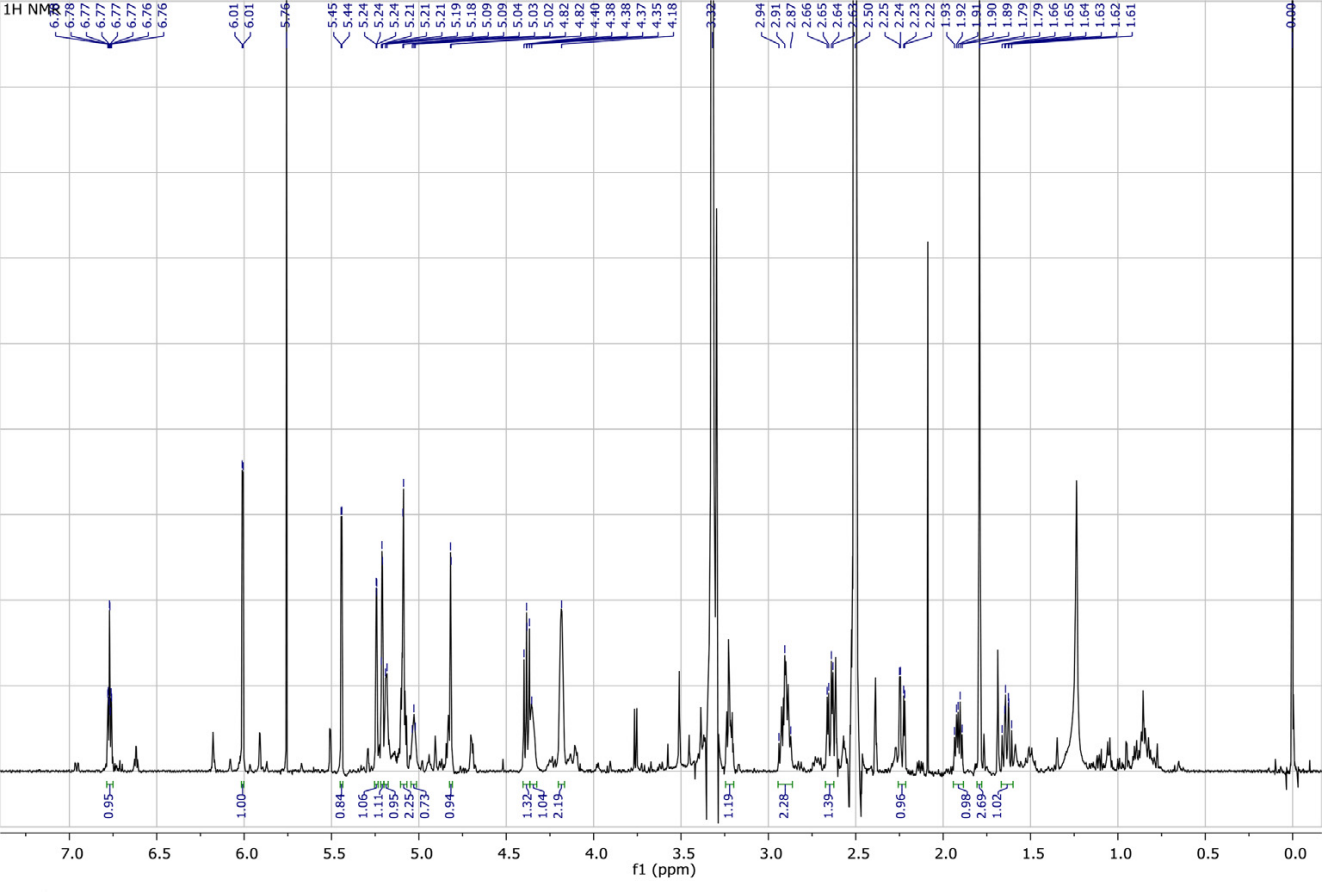
^1^H NMR (DMSO-*d*_*6*_) spectrum of compound **13**.

**Fig. 24:**
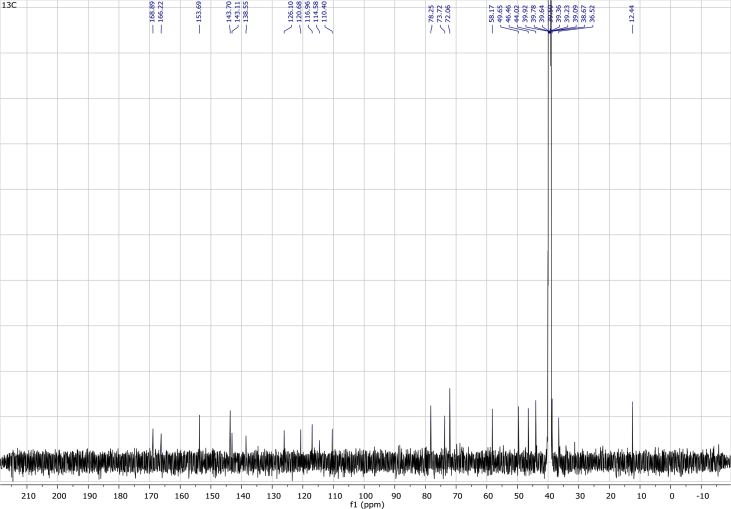
^13^C NMR (DMSO-*d*_*6*_) spectrum of compound **13**.

**Fig. 25:**
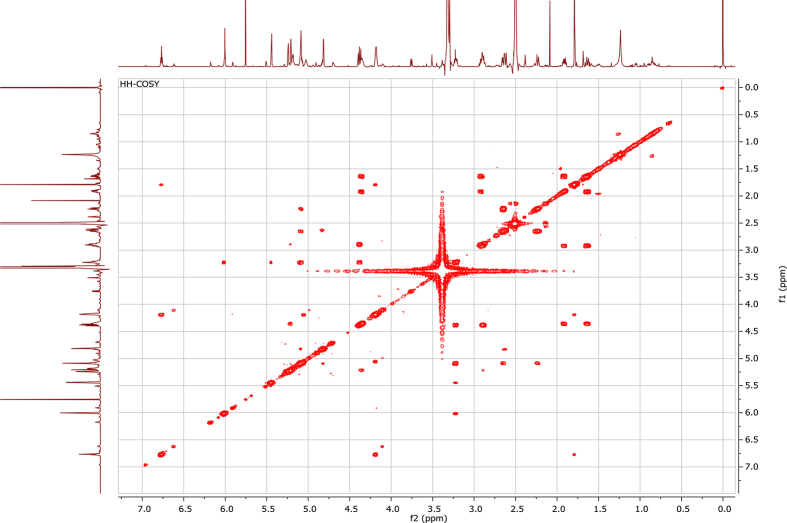
HH-COSY (DMSO-*d*_*6*_) spectrum of compound **13**.

**Fig. 26:**
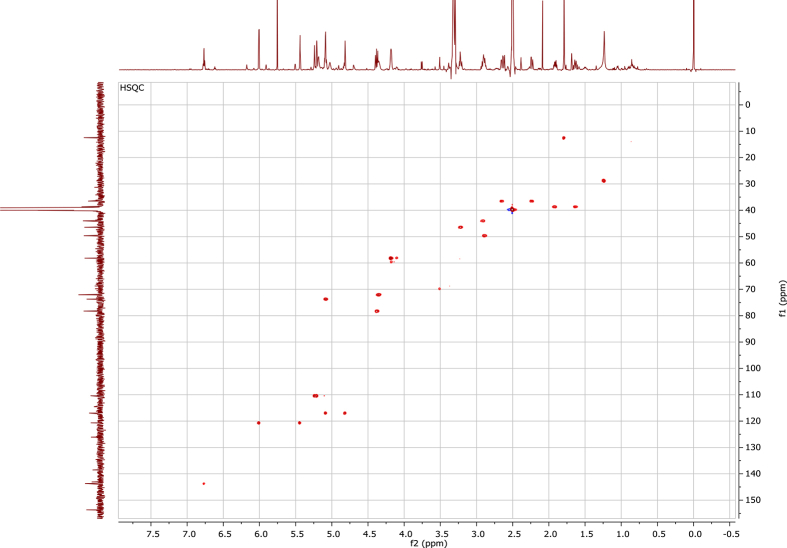
HSQC (DMSO-*d*_*6*_) spectrum of compound **13**.

**Fig. 27:**
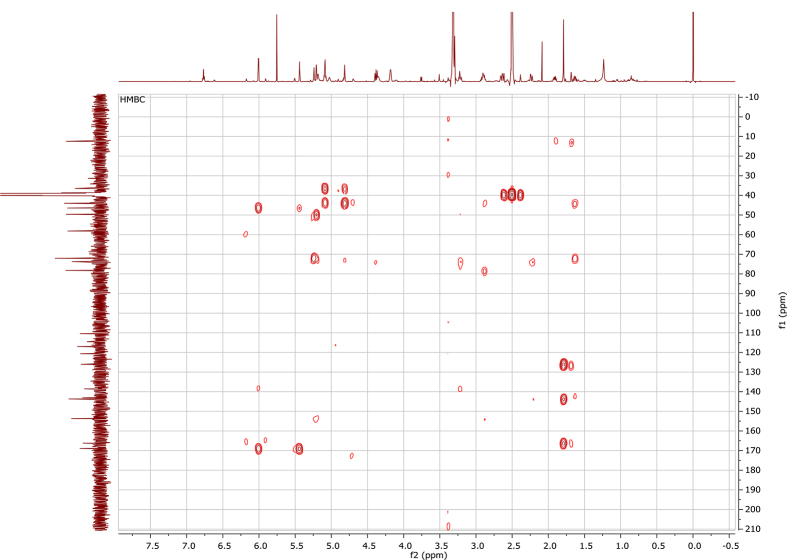
HMBC (DMSO-*d*_*6*_) spectrum of compound **13**.

**Fig. 28:**
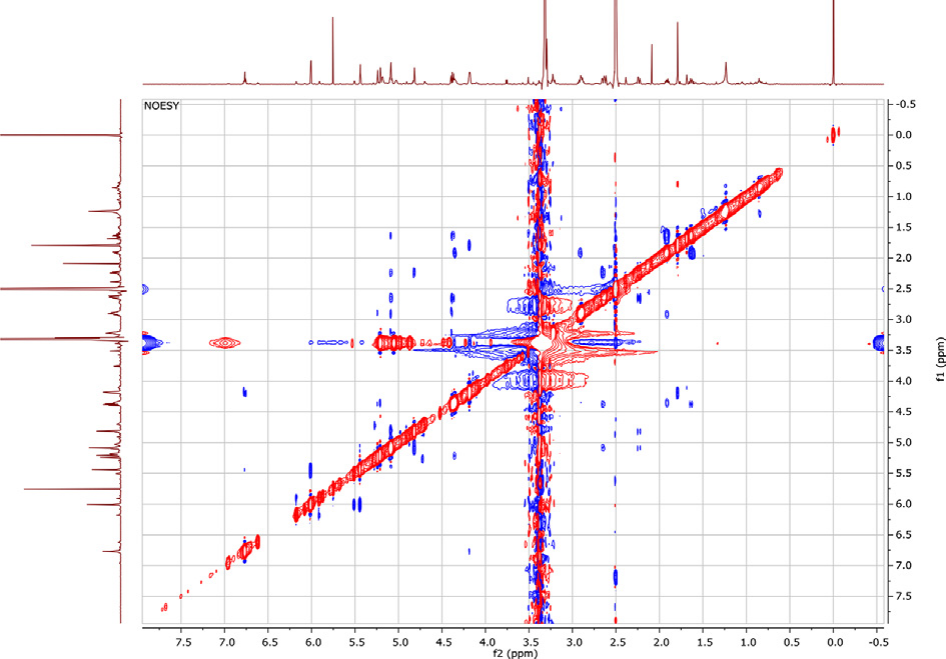
NOESY (DMSO-*d*_*6*_) spectrum of compound **13**. The intensity of the spectrum was tuned to show the cross-peaks due to the dipolar couplings (blue).

In the course of our investigations [1], it emerged that hydroxyl groups (OH) are best detected in the NOESY spectrum, which usually serves for the stereochemical studies (e.g. [2], [3], [4]). In this type 2D NMR spectrum the OH signals are recognized by the chemical exchange cross-peaks with the solvent residual water signal. Those peaks have the same phase as diagonal (positive) but opposite to all other resonances with dipolar couplings (negative) [5]. The correlations (Fig. 1) observed in the NOESY spectra (Fig. 2, Fig. 3, Fig. 4, Fig. 5) and other 2D NMR spectra for the signals of the OH groups occurring in three sesquiterpene lactones: a germacranolide (8α-(*E*)*-*4′-hydroxysenecioyloxy-9α-hydroxyparthenolide) (**5**) and two guaianolides (cynaropicrin and desacylcynaropicrin 8α-(*Z*)-(4′-hydroxy-2′-methyl)butenoate) (**12**, **13**), are presented. The set of 1D and 2D NMR spectra of compound **5** recorded in CDCl_3_ (Fig. 6, Fig. 7, Fig. 8, Fig. 9, Fig. 10) and in DMSO-*d*_*6*_ (Fig. 11, Fig. 12, Fig. 13, Fig. 14, Fig. 15, Fig. 16) and those of compounds **12** (Figs. Fig. 17, Fig. 18, Fig. 19, Fig. 20, Fig. 21, Fig. 22) and **13** (Fig. 23, Fig. 24, Fig. 25, Fig. 26, Fig. 27, Fig. 28) recorded in DMSO-*d*_*6*_, is also provided in this article. The typical NMR data for these compounds, including OH resonances, like chemical shifts, multiplicity and coupling constants, are reported in the main article [1]. Generally, as can be seen in the case of compound **5**, two OH groups resonated much more downfield in DMSO-*d*_*6*_ (range 5.2–5.8 ppm) than in CDCl_3_ (range 2.0–2.6 ppm). The chemical exchange cross-peaks between those OH groups were also observed.

## 2 Experimental design, materials and methods

The NMR spectra were recorded on a Bruker Avance 600 instrument using 600 MHz and 150 MHz frequencies for hydrogen nuclei (^1^H) and carbon nuclei (^13^C), respectively, and tetramethylsilane (TMS) as internal standard. The spectra were obtained for CDCl_3_ or DMSO-*d*_*6*_ solutions at 298 K. No special pulse programmes were used. It should be emphasized, that NMR measurements were made in aprotic solvents without the addition of solvents causing deuterium exchange, like CD_3_OD, D_2_O or TFA-*d* (CF_3_COOD), but containing residual water, what is crucial for the hydroxyl group detection in the NOESY spectra. Therefore, those solvents must not be dried to remove the mentioned water completely. To recognize the ^1^H signals of hydroxyl groups two approaches were utilized – the direct and indirect one. In the direct approach the NOESY spectrum was examined for the presence of proton signals exhibiting cross-peaks with the residual water signal. In the indirect approach, the HSQC spectrum was examined for the ^1^H signals lacking cross-peaks with carbon atom signals, i.e. without one-bond ^1^H–^13^C couplings.
